# A pilot study of Brazilian-adapted MHFA-informed program

**DOI:** 10.3389/fpsyt.2026.1794845

**Published:** 2026-07-22

**Authors:** K. N. Mendes, S. Scotti Requena, A. A. Loch, W. Rössler

**Affiliations:** 1 Laboratory of Neuroscience (LIM-27), Institute of Psychiatry, Hospital das Clínicas, Faculdade de Medicina, Universidade de São Paulo (HCFMUSP), São Paulo, SP, Brazil; 2 Centre for Mental Health and Community Wellbeing Melbourne School of Population and Global Health The University of Melbourne, Melbourne, VIC, Australia; 3 Department of Psychiatry and Psychotherapy, Charité University of Medicine, Berlin, Germany; 4 Department of Psychiatry and Psychotherapy, University of Zurich, Zurich, Switzerland

**Keywords:** Brazil, mental health first aid, mental health literacy, psychosocial intervention, stigma

## Abstract

Mental disorders represent a growing global burden, with particularly large treatment gaps in low- and middle-income countries such as Brazil. Educational initiatives like Mental Health First Aid (MHFA) seek to improve mental health literacy and reduce stigma among lay populations. This pilot study evaluated the feasibility, applicability, and preliminary findings of a Brazilian-adapted MHFA-informed program based on culturally adapted Brazilian MHFA guidelines. A total of 124 employees from three workplaces (two non-profit organizations and one private company) were allocated into intervention and control groups according to institutional operational feasibility, participant registration order, and workplace scheduling demands. The Brazilian-adapted MHFA-informed program consisted of five 1-hour sessions covering depression, psychosis, suicide, trauma, and alcohol-related problems; the control group received Ministry of Health educational materials. Outcomes included attitudes toward mental illness (EMO-56) and perceived stigma (PDDQ), assessed at baseline (T1) and post-intervention (T2). No significant group differences were observed, although both groups showed improvements over time, particularly in the Policy and Structural Assistance dimension (p<0.001). Participants with higher baseline knowledge demonstrated more pronounced improvements over time, and those with self-reported mental illness exhibited more positive attitudes and less stigma. These findings support the feasibility and cultural applicability of a Brazilian-adapted MHFA-informed program in workplace settings. Although limited by sample size and participant attrition, the study highlights the potential relevance of culturally adapted psychoeducational interventions for promoting mental health knowledge and reducing stigma in Brazilian contexts. Future controlled studies with larger samples are needed to further evaluate these approaches.

## 1 Introduction

The growing prevalence of mental disorders worldwide has placed significant pressure on health systems, highlighting the disparity between the demand for specialized services and the availability of adequate care (1). The impacts of these conditions are wide-ranging and complex, affecting not only individuals but also their families and communities (2). People experiencing mental health problems often face difficulties in several areas of life, including work, education, and interpersonal relationships. They are also at increased risk of developing chronic conditions such as cardiovascular disease, hypertension, stroke, diabetes, metabolic syndrome, and obesity (3, 4). According to the World Health Organization, only a small proportion of individuals with mental health conditions receive adequate treatment, often due to limited professional training and the absence of clear care strategies. In low- and middle-income countries (LMICs), such as Brazil, the treatment gap is particularly severe, with estimates indicating that between 76% and 85% of individuals with severe mental health conditions receive no form of treatment (5, 6). Misinformation regarding appropriate mental health support practices may lead to inadequate interventions or neglect, further exacerbating the suffering of individuals at risk of mental illness (4).

Limited knowledge regarding how to support individuals with mental health problems represents a significant public health concern affecting both people living with these conditions and the communities in which they live. Insufficient technical and educational training—both within health institutions and among the general population—may contribute to stigma and prejudice, making access to appropriate care more difficult (7–9). When individuals do not understand the nature of mental health problems, they may adopt inappropriate attitudes, such as blaming people for their condition or minimizing the severity of their symptoms (4). As a result, stigma and discrimination continue to persist in many societies, preventing individuals from seeking help due to fear of judgment and social exclusion. The stigma associated with mental health conditions remains one of the main barriers to accessing adequate care (10). In this context, educational strategies and training programs play an important role in promoting a culture of empathy and evidence-based support for individuals experiencing psychological distress. Studies suggest that educational interventions, including mental health training for lay populations, may reduce stigma and improve the ability to identify and respond early to mental health crises (11–13). Therefore, investing in education and awareness initiatives is essential for building a more inclusive society prepared to provide effective support to people experiencing mental health problems.

As such, Mental Health First Aid (MHFA) was developed in Australia in 2000 with the aim of training members of the general public to provide initial support to individuals experiencing mental health problems or crises (14). MHFA is an educational approach designed to teach individuals how to recognize signs of mental health problems or crises in others and how to provide initial support and guidance until appropriate professional help is obtained or the crisis is resolved (15). It encompasses assisting individuals who are developing a mental health problem, experiencing worsening symptoms, or undergoing a mental health crisis. The guidelines aim to teach people how to identify early signs and symptoms of mental health conditions or crises, provide immediate support, and encourage individuals to seek appropriate professional treatment. MHFA has been adapted for different populations and contexts, including adolescents, young adults, older adults, Aboriginal peoples, higher education students, financial advisors, and pharmacists, among others (14). Part of the initiative consists of guidelines developed through a Delphi consensus process involving people with lived experience and mental health professionals with expertise in the field. These guidelines address mental health conditions such as depression, anxiety, psychosis, alcohol-related problems, and crisis situations (16) (17). They are intended to provide evidence- and consensus-based recommendations regarding how members of the general public can recognize mental health problems, approach individuals in distress, offer initial support, and encourage appropriate professional help-seeking. The guidelines are culturally adaptable and have been translated and modified for use across different countries and sociocultural contexts. They also serve as a conceptual and practical foundation for MHFA-informed educational and training programs, guiding both the educational content and the supportive actions addressed during training activities.

To date, MHFA has been implemented in more than 22 countries, with over 2 million people trained worldwide (16). Meta-analytic studies indicate that MHFA is associated with several consistent benefits, including reduced stigma, increased mental health knowledge, improved recognition of mental health problems, and greater confidence in providing support to individuals experiencing psychological distress. These positive effects have been observed across different cultural, social, and economic contexts, reinforcing the potential relevance of mental health first aid initiatives for promoting mental health literacy and strengthening community support networks (16, 18–22). In Brazil, international MHFA guidelines were translated and culturally adapted into Portuguese. To date, five sets of guidelines have been adapted to the Brazilian context, addressing traumatic situations, depression, suicide, psychosis, and alcohol-related problems (23–27). Brazilian guidelines, a brief MHFA-informed psychoeducational program was developed for implementation in workplace settings. The program was structured using educational content and support strategies derived from the adapted guidelines while considering Brazilian sociocultural characteristics and operational workplace demands.

Based on these culturally adapted Brazilian guidelines, a pilot psychoeducational intervention was developed for implementation in workplace settings. The program was informed by MHFA principles and educational content while being adapted to the Brazilian sociocultural context and operational realities. Rather than replicating the official MHFA training model, the present study sought to explore the feasibility, applicability, and preliminary effects of a Brazilian-adapted MHFA-informed program implemented in a real-world workplace context. The program retained key MHFA principles related to recognition of mental health problems, initial support, stigma reduction, and encouragement of professional help-seeking, while being adapted to the Brazilian workplace context.

The aim of the current study was to develop and conduct a preliminary evaluation of a brief psychoeducational program based on previously adapted Brazilian MHFA-informed guidelines.

## 2 Methods

### 2.1 Study design

This study employed a quasi-experimental pilot design with intervention and active comparison groups. Participants were allocated according to institutional operational feasibility, participant registration order, and workplace scheduling demands rather than through formal randomization. The study aimed to conduct a preliminary evaluation of the feasibility, applicability, and potential effects of a Brazilian-adapted MHFA-informed psychoeducational program implemented in workplace settings.

### 2.2 Sample

Of the 124 participants enrolled at baseline (T1), 80 completed the post-intervention assessment (T2), corresponding to a retention rate of 64.5%. Specifically, 47 participants from the intervention group and 33 from the control group completed T2 and were included in the primary inferential analyses. Due to substantial attrition at follow-up, T3 assessments were considered exploratory and were not included in the main longitudinal analyses. A total of three workplaces participated in this pilot study: two non-profit organizations and one private company, all located in the metropolitan area of São Paulo, Brazil. These organizations were identified through internet searches and contacted by e-mail, followed by telephone calls. Institutions were eligible if they employed lay adult populations aged 18 years or older, meaning staff who did not routinely work with mental health-related care or services.

Before the course began, the administrators of each institution provided employees with information regarding the program, including its theme, schedule, and duration. Individuals who expressed interest were invited to register through an online registration form, which confirmed their willingness to participate in the study. Upon registration, participants received detailed written information about the study—including its voluntary nature, confidentiality procedures, and the right to withdraw at any time without penalty—and were asked to provide electronic informed consent prior to participation.

A total of 124 employees from the three participating organizations were enrolled in the study. Retention rates were 64.5% from T1 to T2 (n=80), 42.7% from T2 to T3 (n=52), and 27.4% from T1 to T3 (n=34). Due to substantial attrition at follow-up, T3 data were considered exploratory and were not included in the primary inferential analyses.

The present study was approved by the Research Ethics Committee of the Hospital das Clínicas, Faculdade de Medicina, Universidade de São Paulo (CAPPesq), under CAAE registration number 20017419.5.0000.0068. Due to the COVID-19 pandemic context, most study procedures and data collection activities were conducted online. However, intervention delivery format varied according to institutional feasibility, with one organization participating in person and two organizations participating remotely. Participants were informed about the study objectives and procedures prior to participation, and electronic informed consent was obtained before data collection.

### 2.3 Procedures

Once participants had registered, groups were organized in collaboration with institutional administrators according to the number of registrations, institutional availability, and workplace demands. Participants were allocated to intervention and control groups considering registration order and operational workplace needs rather than formal randomization procedures. For both groups, five sessions were conducted, each lasting approximately one hour and scheduled on different days of the week according to a previously established timetable. One session was dedicated to each thematic topic covered in the program. Sessions included visual presentations (using Datashow resources), theoretical content, and case examples related to mental health problems and crisis situations. Because the intervention was delivered within participants’ workplace routine and during working hours, attendance was incorporated into institutional activities, and no minimum attendance criterion was established for inclusion in the analyses.

The intervention implemented in this study was a Brazilian-adapted MHFA-informed psychoeducational program developed based on previously translated and culturally adapted MHFA guidelines for the Brazilian context. The program was structured by the research team using adapted materials and psychoeducational content derived from prior Delphi studies conducted in Brazil. Although informed by MHFA principles and educational materials, the intervention did not correspond to the official certified international MHFA training model. Instead, the adapted guidelines were used to organize the educational content, case examples, and mental health support strategies addressed during the intervention. The program was designed as a brief pilot intervention adapted to workplace settings and operational constraints observed during the COVID-19 pandemic period.

Although the program did not correspond to the official certified MHFA training model, it retained several core MHFA principles reflected in the guidelines that had previously undergone translation, cultural adaptation, and contextualization for the Brazilian setting. These principles included improving recognition of common mental health problems and crisis situations, increasing mental health literacy, promoting supportive and non-stigmatizing responses, encouraging appropriate professional help-seeking, and providing guidance on how members of the public can offer initial support to individuals experiencing mental health difficulties. The translated and culturally adapted guidelines served as the foundation for the development of the educational content, case examples, and support strategies addressed throughout the program.

The sessions were conducted exclusively by the principal researcher, a psychologist trained in the study protocol and in the adapted Brazilian MHFA-informed guidelines.

Data collection was conducted at three timepoints: baseline before the intervention (T1), immediately after completion of the program (T2), and one month after the intervention (T3). All questionnaires were administered online using electronic forms, regardless of whether participants attended sessions in person or remotely. Therefore, differences in intervention delivery format did not substantially affect data collection procedures across groups.

In the intervention group, the adapted MHFA-informed guidelines addressing traumatic situations, depression, psychosis, suicide, and alcohol-related problems were presented. The educational content included: (a) an overview of mental health conditions and crisis situations; (b) epidemiological information; (c) presentation and discussion of the adapted guidelines; and (d) guidance regarding referral to mental health and healthcare services. In the control group, previously selected educational booklets developed by the Brazilian Ministry of Health (28) were presented, covering mental health topics such as traumatic situations, depression, schizophrenia, suicide, and alcohol and drug-related problems. Therefore, the control condition represented an active psychoeducational comparison group rather than a passive or no-intervention control condition.

The research instruments were administered online at all assessment stages. Due to substantial attrition at follow-up, T3 assessments were treated as exploratory and excluded from the primary inferential analyses. Data were collected using Google Forms, which ensured participant anonymity and minimized potential researcher influence on responses. The program was delivered using different formats according to institutional feasibility. The private organization conducted all sessions in person, whereas the two non-profit organizations delivered all training sessions remotely.

### 2.4 Outcomes

The primary outcomes of interest were changes in stigma-related attitudes and perceptions toward mental illness assessed using the Opinions about Mental Illness Scale (EMO-56), and the Perceived Devaluation-Discrimination Questionnaire (PDDQ). The study also explored associations between baseline self-reported mental health knowledge and changes over time across specific attitudinal domains. Assessments were conducted at three timepoints: T1 (baseline/pre-intervention), T2 (immediately after completion of the program), and T3 (one-month follow-up).

### 2.5 Instruments

The instruments included a sociodemographic questionnaire, along with measures assessing knowledge about mental health and stigma-related attitudes toward mental illness. Additional variables included: (1) self-reported experience of mental health symptoms (yes/no); (2) presence of mental health symptoms in a family member (yes/no); and (3) self-reported mental health knowledge.

Self-reported mental health knowledge was assessed using a single Likert-scale item and was treated as an exploratory indicator rather than a comprehensive measure of mental health literacy. Responses were recorded on a 5-point Likert scale ranging from 1 (low levels, indicating limited knowledge or fewer symptoms) to 5 (high levels, indicating extensive knowledge or greater symptom presence).

Two other scales were also used: the Opinions about Mental Illness Scale (EMO-56) (29), and the Perceived Devaluation-Discrimination Questionnaire (PDDQ) (30) The EMO-56 is a self-report questionnaire originally developed in Portuguese and validated in Brazilian samples. It comprises 56 items, each rated on a 5-point Likert scale ranging from 1 (“strongly disagree”) to 5 (“strongly agree”). Several items are reverse-scored to control for response bias. The total score is obtained by summing all item responses after appropriate reverse coding, with lower scores reflecting more negative or stigmatizing attitudes toward mental illness. The scale also allows for subscale analyses across dimensions such as *conception of mental illness, perception of the mentally ill person, family care, nurse’s role, and policy and structural assistance.* Scoring procedures followed the validation study by Osinaga and Furegato (31).

The EMO-56 is a Brazilian instrument developed to assess attitudes and stigma-related perceptions regarding mental illness across multiple dimensions, including authoritarianism, social restriction, interpersonal etiology, mental hygiene ideology, benevolence, minority view, family care, policy and structural assistance, and interpersonal discomfort. Example items include beliefs regarding the role of family support in mental health care and perceptions about institutional and community assistance available for individuals with mental illness. Previous studies have applied the instrument in Brazilian populations to assess stigma-related attitudes toward mental illness. Although originally developed in healthcare-related contexts, the “Nurse’s Role” dimension was retained in the present study because it reflects broader perceptions regarding professional support, care responsibilities, and attitudes toward mental health assistance, which were considered relevant for workplace psychoeducational contexts.

The Perceived Devaluation-Discrimination Questionnaire (PDDQ) was used to assess perceived stigma and coping responses related to mental illness. The extended version employed in this study includes three subscales: (1) Perceived Devaluation-Discrimination (12 items), which measures the extent to which respondents believe that people generally devalue individuals with mental illness; (2) Secrecy (9 items), which evaluates the tendency to conceal one’s mental health problems to avoid stigma; and (3) Withdrawal (8 items), which assesses avoidance of social interactions as a protective response to anticipated rejection. All items are rated on a 5-point Likert scale ranging from 1 (“strongly agree”) to 6 (“strongly disagree”). Appropriate items are reverse-scored, and higher scores indicate stronger perceived stigma or greater use of secrecy and withdrawal strategies. Subscale and total scores were calculated by averaging the corresponding items after reverse coding, following procedures described by Link et al. (30) and Corrigan et al. (32), and previously applied in Brazilian validation (33, 34).

The instruments employed in the present study had previously been adapted and/or applied in Brazilian contexts. Given the exploratory and pilot nature of the study, these instruments were selected to assess attitudes, perceptions, and self-reported knowledge related to mental health in workplace settings. Internal consistency for the current sample was assessed using Cronbach’s alpha coefficients: EMO56, 0.668 for T1 and 0.788 for T2; PDDQ, 0.813 for T1, and 0.846 for T2.

### 2.6 Statistical analysis

Descriptive analyses were carried out to characterize the sociodemographic profile of the intervention and control groups. Data were described using absolute and relative frequencies for categorical variables and means with standard deviations for continuous variables. Baseline comparisons between the intervention and control groups were conducted prior to the intervention to identify potential group differences in sociodemographic and study variables. Statistically significant differences were assessed using chi-square tests for categorical variables and Student’s t-tests or ANOVA for continuous variables. Given the pilot and exploratory nature of the study, no formal *a priori* sample size calculation was performed.

Changes in outcome scores from baseline to follow-up were examined using repeated-measures general linear models (GLMs). Time was specified as a within-subject factor with two levels, baseline and follow-up, and group was entered as a between-subject factor comparing the intervention and control groups. To reduce the risk of overfitting given the modest sample size, the primary models were adjusted only for age, sex, and education. The primary effect of interest was the time × group interaction, which tested whether changes over time differed between the intervention and control groups. Fully adjusted exploratory models are given in **Supplementary Tables S1** and **S2**.

Estimated marginal means were calculated for each group at each time point, with covariates fixed at their sample means. Pairwise comparisons were adjusted using the Bonferroni correction. Because multiple outcomes were examined, false discovery rate correction (FDR) using the Benjamini–Hochberg procedure was applied to the family of primary time × group interaction tests. Effect sizes were reported as partial eta squared. Model assumptions were assessed using Box’s M test for equality of covariance matrices, Levene’s test for equality of error variances, and Mauchly’s test of sphericity. Because only two time points were included, sphericity was not a substantive concern. Statistical significance was set at p <.05. Eight models were thus constructed, each corresponding to one dependent variable: *conception of mental illness*, *perception of the mentally ill person, family care, nurse’s role, and policy and structural assistance* (EMO-56), as well as *discrimination, secrecy, and withdrawal* (PDDQ).

Primary analyses were restricted to T1 and T2 assessments due to the limited number of participants completing T3 follow-up measures. Analyses were conducted using complete-case data for participants with available T1 and T2 assessments, and no imputation procedures were performed for missing data. All analyses were conducted using SPSS software version 20 for MacOS.

## 3 Results

**Figure 1:**
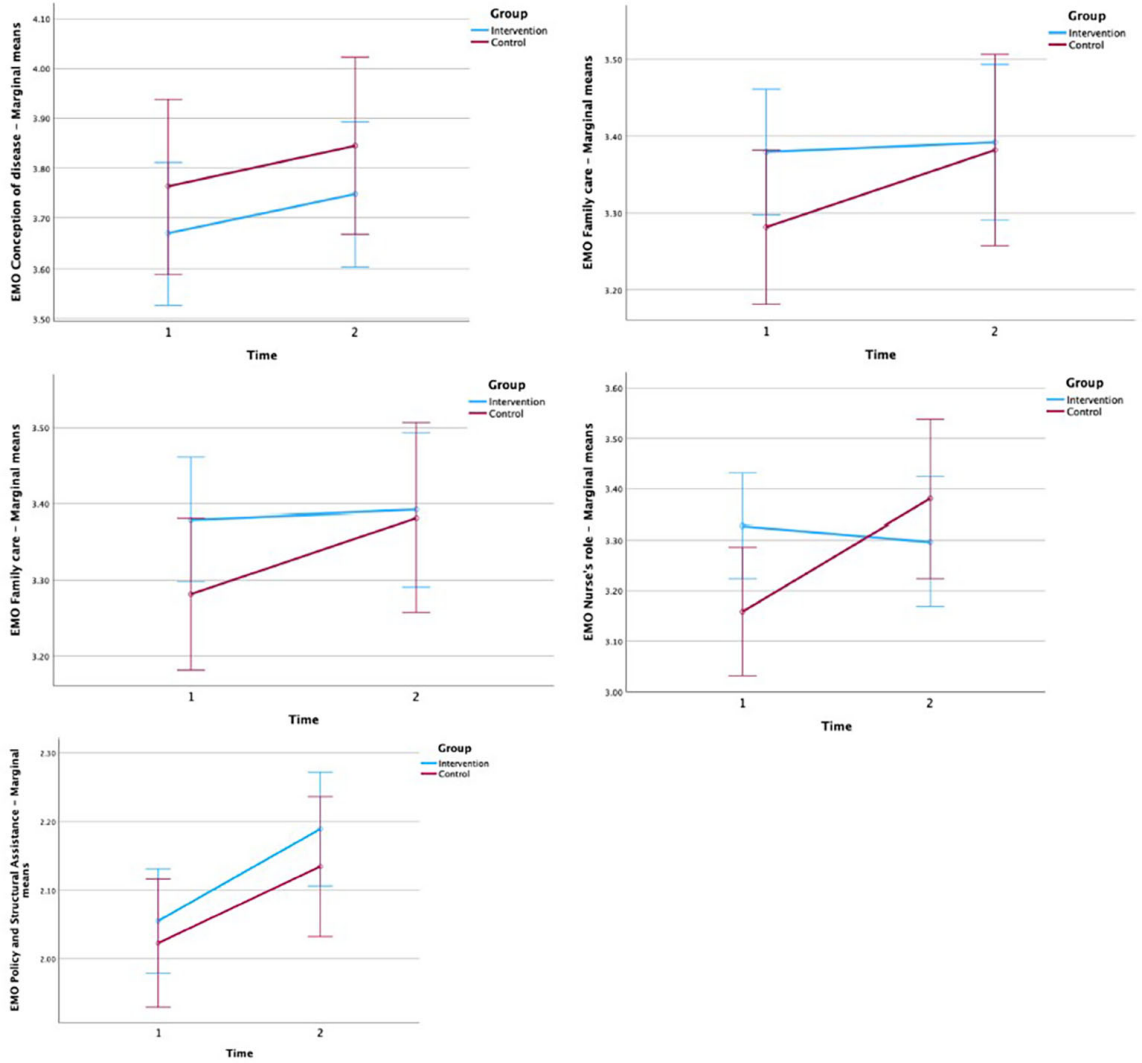
Estimated marginal means of EMO-56 scores across groups at two time points (T1 and T2). Error bars represent 95% confidence intervals. Higher scores indicate better/less stigmatizing opinions.

Participant retention across study phases is described in **Figure 1**. Of the 124 participants initially enrolled, 80 completed the T2 assessment and were included in the primary analyses (47 intervention; 33 control). Retention from T1 to T2 was 67.1% in the intervention group and 61.1% in the control group. Although T3 follow-up data were collected, retention at this stage was substantially lower, and these assessments were treated as exploratory. Due to the largely remote and pragmatic characteristics of the study during the COVID-19 pandemic period, reasons for participant dropout were not systematically collected.

**Table 1** shows the sociodemographic characteristics of the sample. Females represented the majority in both groups (85.1% in the intervention group and 81.8% in the control group). Mean age was significantly higher among control participants (mean=37.2, SD = 9.7) compared to participants in the intervention group (mean=32.9, SD = 11.4) (p=0.043). Most participants were single (51% in the intervention group and 45% in the control group) and had completed higher education (63% vs. 57%, respectively). Regarding state of residence, most participants in both groups lived in the state of São Paulo; however, the control group included significantly more participants from other Brazilian states (p<0.001). The significant difference observed for state of residence reflected the fact that one group was composed exclusively of participants from São Paulo state, whereas the other group included a small proportion of participants from other Brazilian states.

**Table 1:** Baseline sociodemographic characteristics of participants included in the primary T1–T2 analyses (n=80).

	Intervention	Control	p-value
Sex (female, n, %)	40 (85,1%)	27 (81,8%)	0,983
Age (mean, SD)	32,9 (11.4)	37.2 (9.7)	0,043*
Marital Status (singles, n, %)	24 (51.1%)	15 (45.5%)	0,953
Education (graduates, n, %)	30 (63.8%)	19 (57.5%)	0,644
State (SP, n, %)	47 (100%)	25 (75.8%)	<0,001***
Monthly family income (BRL 2,000–3,000)	17 (36.2%)	6 (18.2%)	0,140

“Of the 124 participants initially enrolled, 80 completed the T2 assessment and were included in the primary analyses”.

Pearson's X^2 test of association and U-Mann Whitney test of comparison between independent groups. ¹ Fisher's exact test. ASIMID = ... *p < 0.05, ***p < 0.00.1.

**Table 2** shows the results of the questionnaire regarding mental health and mental illness. In both groups, most participants reported not experiencing symptoms of mental illness themselves (55% in the intervention group and 75% in the control group). However, the majority of participants reported recognizing symptoms of mental illness in a family member (80% vs. 63%, respectively). Most participants rated their knowledge about mental health as 3 or below on a 5-point scale. None of the observed differences between groups were statistically significant.

**Table 2:** Answers to the questionnaire on mental health (n=80).

		Intervention		Control		
		N	%	N	%	p-value
Symptoms of mental illness	Yes	21	44.70%	8	24.20%	0,09
	No	26	55.30%	25	75.80%	
Family member with symptoms of mental illness	Yes	38	80.90%	21	63.60%	0,12
	No	9	19.10%	12	36.40%	
Knowledge of mental health	1	6	12.80%	9	27.30%	0,33
	2	17	36.20%	8	24.20%	
	3	20	42.60%	11	33.30%	
	4	3	6.40%	3	9.10%	
	5	1	2.10%	2	6.10%	

Pearson's X^2 test of association and U-Mann Whitney test of comparison between independent groups. 1 Fisher's exact test. ASIMID = ...

Repeated-measures general linear models (GLMs) adjusted for age, sex, and education were used to examine changes in each outcome from baseline to follow-up (**Supplementary Tables S1**). The primary effect of interest was the time × group interaction. Across the eight outcomes, there was no robust evidence that changes over time differed between the intervention and control groups after correction for multiple testing. The only nominally significant time × group interaction was observed for the nurse role domain, F(1, 74)=5.130, p=0.026, ηp²=0.065. However, this effect did not remain significant after false discovery rate correction, q=0.208. Moreover, adjusted marginal means indicated that nurse role scores remained essentially stable in the intervention group, whereas they increased in the control group. Therefore, this result was interpreted as exploratory and not as evidence of a beneficial intervention effect. For the policy/structural domain, there was a significant overall increase from baseline to follow-up in the estimated marginal means; however, the time × group interaction was not significant, F(1, 74)=0.376, p=0.542, ηp²=0.005, indicating that this increase did not differ between groups. No significant time × group interactions were observed for conception of disease, perception of disease, family care, stigma, secrecy, or withdrawal. Overall, the analyses did not provide evidence that the intervention produced differential improvements in the assessed outcomes compared with the control condition.

Model assumptions were generally acceptable. Box’s M was significant for the nurse role and secrecy models, and Levene’s test at follow-up was significant for withdrawal; therefore, these specific results should be interpreted with caution. Because only two time points were included, sphericity was not a substantive concern.

Although the primary analyses did not provide robust evidence of differential intervention effects, exploratory sensitivity analyses suggested that baseline knowledge may have influenced change (increase) over time in the Policy and Structural Assistance domain (F(1, 69)=4.74, p=0.033, η²p=0.064). This finding should be interpreted cautiously, as it emerged from fully adjusted models (including income level, education level, self-reported mental symptom, mental disorder in the family, marital status and self-reported knowledge about mental illnesses) and was not the primary focus of the study. Nevertheless, it may indicate that participants’ prior knowledge shaped how they interpreted or responded to items related to mental health policies and structural aspects of care. Future studies with larger samples should examine knowledge as a potential moderator of change in attitudes toward mental illness and mental health care.

## 4 Discussion

To date, this was the first pilot study evaluating a Mental Health First Aid (MHFA)-informed program in Brazil. Studies of this nature are important for the development of evidence-based strategies, contributing to professional training and expanding access to effective mental health interventions, as well as informing public policies. In addition, research of this kind may help adapt international guidelines to the Brazilian context, promoting more contextualized and culturally appropriate approaches.

**Figure 2:**
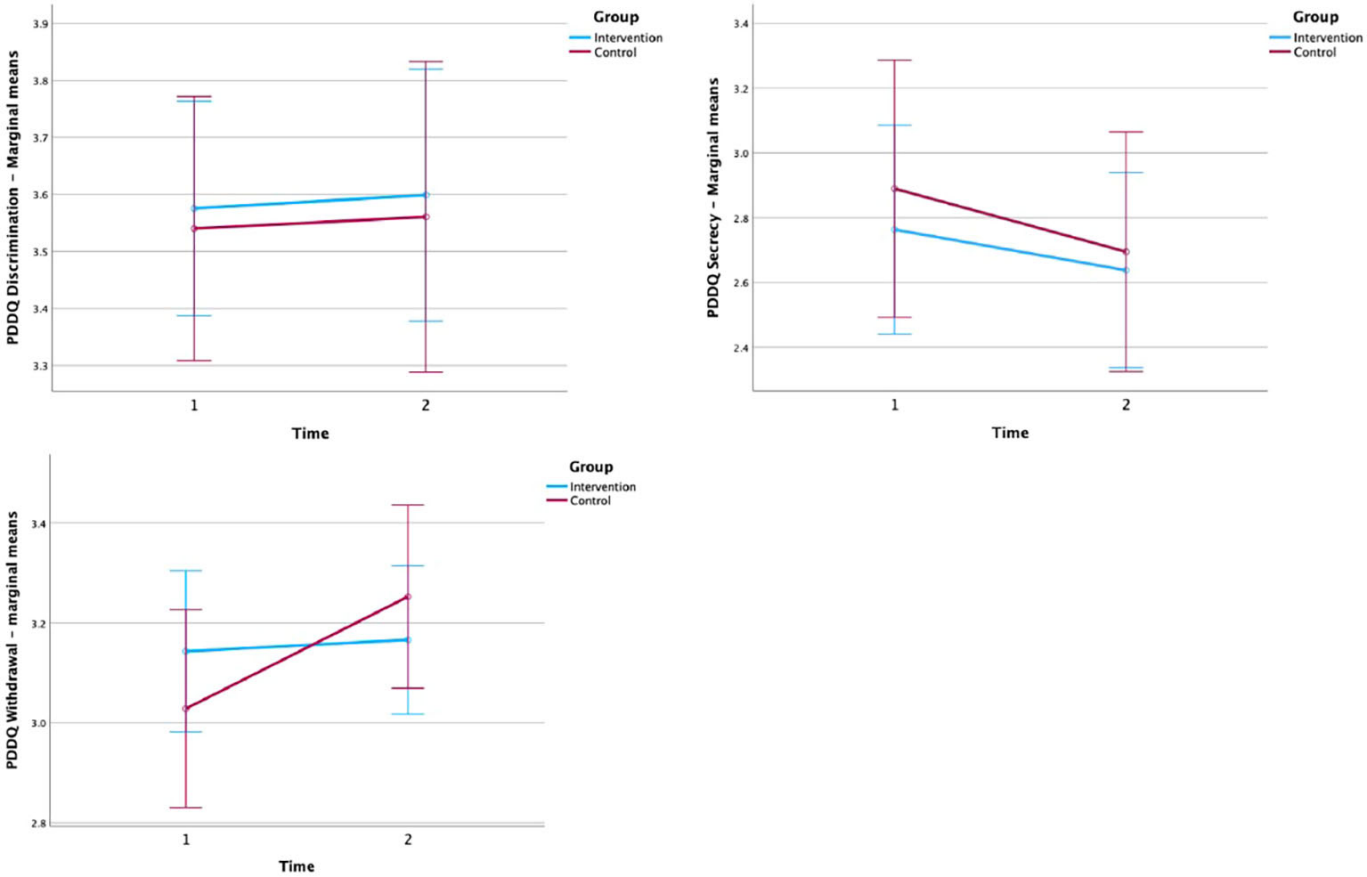
Estimated marginal means of stigma scores (PDDQ) across groups at two time points (T1 and T2). Error bars represent 95% confidence intervals. Higher scores indicate more perceived stigma.

The study findings suggest that participation in both psychoeducational conditions was associated with improvements over time in some dimensions related to mental health attitudes and stigma. However, no statistically significant differences between the intervention and control groups were observed. The use of an active control condition based on Ministry of Health educational materials may have contributed to similar improvements across groups, suggesting that exposure to structured psychoeducational content itself may positively influence self-reported mental health knowledge and stigma-related attitudes. In this sense, the present study may be more appropriately interpreted as a comparison between two psychoeducational mental health approaches rather than between an intervention and a no-treatment condition. A significant effect of Time and self-reported knowledge on mental disorders was observed for the Policy and Structural Assistance dimension, indicating improvement between T1 and T2. This suggests that individuals with greater baseline knowledge demonstrated more pronounced improvements over time, as illustrated in **Figure 2**.

Another important aspect concerns the nature of the observed changes in attitudes and stigma-related dimensions. Although psychoeducational interventions may contribute to greater awareness and more favorable responses regarding mental health, it is not possible to determine whether these changes reflect deeper subjective transformations or primarily cognitive alignment with socially desirable discourses presented during the program. This distinction is particularly relevant in workplace settings, where institutional norms and expectations may influence how participants respond to mental health-related content. Therefore, the present findings should be interpreted cautiously, recognizing that changes in self-reported attitudes do not necessarily correspond to long-term changes in personal beliefs, behaviors, or interpersonal practices. Future studies incorporating qualitative methods and longer follow-up periods may help clarify the subjective sustainability and experiential impact of these interventions.

Findings revealed comparable results between both psychoeducational approaches. This equivalence suggests that the program may convey psychoeducational content in a practical and accessible manner, meeting the needs of a diverse audience. It also indicates that the program shares relevant characteristics with well-established educational strategies in Brazil, such as the educational booklets developed by the Ministry of Health. On the other hand, these findings differ somewhat from the broader literature, which generally reports superior outcomes for first aid initiatives when compared with control groups (13, 35). This divergence may be explained by the fact that previous studies did not employ another mental health literacy intervention as a comparator, as was done in the present study. The present methodology, by employing an active psychoeducational comparison group, offered several advantages, most notably a more balanced management of participants’ expectations and a stronger comparative framework for evaluating the program relative to established educational strategies in Brazil. This approach also helped minimize potential bias, thereby strengthening the validity and reliability of the study’s conclusions (36, 37).

The interaction between Time and self-reported knowledge was observed specifically within the Policy and Structural Assistance dimension. This finding may suggest that participants with greater baseline familiarity with mental health concepts demonstrated more pronounced changes over time in this specific attitudinal domain. However, this effect should be interpreted cautiously, as it was restricted to a single subscale and did not represent a generalized intervention effect across all outcomes (38). In the context of mental health literacy, prior knowledge may enhance cognitive engagement and information integration, enabling participants to connect new content with existing conceptual frameworks (39). Studies have shown that preexisting literacy facilitates deeper processing of educational material, resulting in greater attitudinal and behavioral change (40). In evaluations of other first aid programs, participants with higher baseline literacy or prior exposure to mental health topics often demonstrate stronger post-training gains in confidence and supportive behaviors (16, 41). These findings suggest that the program may function not only as a knowledge-transfer mechanism but also as a knowledge-activation process, consolidating and expanding preexisting conceptual schemas. Furthermore, greater familiarity with mental health issues may reduce defensiveness and stigma, allowing participants to assimilate new information more effectively (42). The moderating role of initial literacy therefore has practical implications for program design. Tailoring interventions according to participants’ baseline knowledge—through differentiated instruction or pre-course assessment—may enhance the applicability and educational benefits of the intervention across heterogeneous groups. It also reinforces the notion that mental health knowledge acquisition is a cumulative and socially embedded process, in which prior exposure, cultural norms, and experiential learning shape how educational content translates into meaningful changes in attitudes and behavior (43).

The literature reiterates the importance of mental health literacy for the early recognition of mental disorders, promoting greater help-seeking behavior and adherence to treatment, reducing hospitalizations, and decreasing stigma at multiple levels (44, 45). In addition, studies emphasize the important role of personal experiences shared by individuals with mental disorders in the stigmatization process, highlighting that their impact may be either positive or negative depending on how these narratives are presented and received by the public (46). Our findings, for instance, showed that participants who reported having a mental disorder demonstrated greater knowledge about mental illness and lower levels of stigma. This highlights the importance of involving different stakeholders, including people with lived experience, in anti-stigma initiatives (47).

Concerning the limitations of the study, we encountered considerable challenges during its implementation, particularly in recruiting companies willing to participate. Although initial interest was high, many organizations later withdrew, citing time constraints. This resulted in significant delays and required intensified efforts to identify suitable sites for data collection. Given the pilot and pragmatic nature of the study, participant allocation was conducted according to institutional operational feasibility, which may have introduced allocation-related bias. Additionally, the modality of intervention delivery differed across institutions, with one organization receiving in-person sessions and two institutions participating remotely. Exploratory comparisons did not suggest substantial differences according to delivery modality; however, because modality overlapped with organizational context, it was not possible to fully disentangle whether the observed effects were associated with the intervention itself, delivery format, or institutional characteristics. This limitation should be considered when interpreting the findings. Interestingly, although the original study design targeted general employees, participating organizations often chose to involve individuals occupying higher hierarchical positions, such as managers and executives, suggesting institutional recognition of the importance of mental health-related topics. Nevertheless, these recruitment difficulties may also reflect broader societal barriers to addressing mental health in workplace settings, including persistent stigma, prejudice, and limited awareness regarding the value of preventive interventions. In this regard, another interesting aspect concerns the baseline characteristics of the sample. Both groups presented relatively high average scores across outcome measures at baseline (T1). It is possible that the institutions agreeing to participate already promoted a culture of dialogue and reflection regarding emotional and mental health-related issues, which may have contributed to participants’ prior familiarity with mental health concepts - a potential selection bias. Although this level of mental health literacy differs somewhat from what is commonly reported in the literature, it reinforces the importance of preventive and empowering approaches aimed at strengthening individuals’ capacity for reflection and autonomy (48). Additionally, mental health literacy was not assessed using a comprehensive validated literacy instrument. Instead, the study relied on a brief self-reported measure of perceived mental health knowledge, which should be interpreted as an exploratory indicator rather than a full operationalization of the broader mental health literacy construct. Participant retention also proved challenging, with a notable dropout rate across the study phases, limiting data analysis and affecting the generalizability of the findings. In addition, the pilot nature of the study and the reduced sample size at follow-up may have limited the statistical power to detect small between-group differences, particularly considering the active psychoeducational comparison condition employed. Possible explanations include work-related demands, perceived lack of personal relevance of the topic, and discomfort discussing sensitive mental health issues. The absence of direct researcher involvement during the follow-up phase may also have contributed to attrition. Dropout rates are commonly observed in workplace-based pilot interventions and may reflect competing occupational demands, limited time availability, and reduced engagement in longitudinal follow-up procedures. Consequently, the one-month follow-up assessments (T3) were treated as exploratory and were not included in the primary inferential analyses. Despite these limitations, the experience underscores the importance of continued investigation - particularly through larger controlled studies - to better evaluate the feasibility, applicability, and potential effectiveness of culturally adapted psychoeducational interventions across diverse professional and organizational contexts.

In summary, this pilot study provides preliminary evidence supporting the feasibility and cultural applicability of a Brazilian-adapted MHFA-informed program in workplace settings. Although no statistically significant differences were observed between groups, both psychoeducational conditions were associated with improvements over time in selected dimensions related to mental health attitudes and stigma. The findings highlight the relevance of culturally adapted psychoeducational approaches and support the continued investigation of mental health literacy interventions in Brazilian contexts through larger and more robust controlled studies.

## Data Availability

The original contributions presented in the study are included in the article/**Supplementary Material**. Further inquiries can be directed to the corresponding author.
